# Effect of Bleaching on the Surface Roughness of Resin Composites Evaluated by Atomic Force Microscopy (AFM)

**DOI:** 10.3390/dj13100470

**Published:** 2025-10-15

**Authors:** Kabas Fadhil, Bassam Karem Amin

**Affiliations:** Department of Conservative Dentistry, College of Dentistry, Hawler Medical University, Erbil 44001, Iraq; kabas.fadhil@den.hmu.edu.krd

**Keywords:** dental restoration, microhybrid composite, nanohybrid composite, surface roughness, zoom bleaching, laser bleaching, AFM

## Abstract

**Background/Objectives**: Composite resins are widely used restorative materials, but their surface properties may be altered by bleaching procedures. This study aimed to compare the effects of two bleaching techniques—light-activated (zoom) and diode laser-activated—on the surface roughness of nanohybrid and microhybrid composites using Atomic Force Microscopy (AFM) for topographic evaluation. **Methods**: A total of 60 composite resin disks were fabricated, with 30 nanohybrid and 30 microhybrid samples. Each type was divided into three subgroups: control, zoom bleaching, and laser bleaching (n = 10 per group). Zoom bleaching employed 40% hydrogen peroxide gel activated by the Philips Zoom system, while laser bleaching used a 940 nm diode laser (QuickLase, Kent, UK) in combination with QuickLase bleaching gel containing approximately 35–40% hydrogen peroxide. Surface roughness parameters (Sa) were measured using AFM, and statistical analysis was performed. **Results**: Both bleaching protocols increased surface roughness compared to controls. Microhybrid composites showed higher roughness after zoom (103.12 ± 19.25 nm) and laser bleaching (106.16 ± 25.21 nm), while nanohybrid composites had lower values after zoom (57.77 ± 13.88 nm) and laser bleaching (78.13 ± 23.29 nm). Significant differences were found between composite types post-bleaching (*p* < 0.001 for zoom; *p* = 0.019 for laser). However, differences between bleaching methods within the same composite type were not significant (*p* > 0.05). **Conclusions**: Both zoom and laser bleaching negatively affect composite surfaces, with laser bleaching showing a greater impact. Nanohybrid composites demonstrated superior resistance to surface alteration, suggesting better clinical durability. These findings are relevant for clinicians when planning restorative treatments in patients likely to undergo bleaching.

## 1. Introduction

Composite resins are among the most prevalent materials in restorative dentistry, valued for their direct-filling application and superior aesthetic properties that mimic natural tooth structure [1]. Continuous evolution in material science has led to the development of composites with varied filler sizes and distributions. A significant advancement is the nanohybrid composite, which integrates nano-sized fillers into a hybrid matrix, aiming to combine the superior mechanical properties of hybrid composites with the high polishability and aesthetics of microfilled composites [2,3].

The clinical performance and longevity of a composite restoration are profoundly influenced by its surface characteristics. A smooth surface is critical, as it directly reduces the risk of fracture, prevents discoloration, and enhances patient comfort [4,5]. Conversely, surface roughness promotes bacterial plaque adhesion and retention, thereby elevating the risks of periodontal disease and secondary dental caries [6].

While some studies report comparable clinical performance between nanohybrid and microhybrid composites [7,8], a systematic review indicates that current evidence remains inadequate to conclusively establish the superiority of one material category over the other in terms of long-term surface smoothness and gloss retention [9].

A significant clinical challenge to the surface integrity of composite restorations is their exposure to chemical agents, particularly high-concentration hydrogen peroxide gels (35–40%) used in professional in-office tooth bleaching procedures. These agents can interact with the organic resin matrix and the filler–resin interface, potentially leading to surface degradation, alteration in mechanical properties, and increased roughness. Furthermore, the bleaching process is often accelerated using various activation methods, such as light-emitting diode (LED) units or lasers, which may contribute differentially to the surface alterations of composite materials [10,11,12,13].

The surface quality of a material can be precisely quantified using non-contact profilometry, with key parameters including Sa (arithmetic mean height) [14].

Therefore, this in vitro study aimed to assess and compare the impact of different professional bleaching protocols on the surface morphology of two distinct composite materials. The rationale for selecting a conventional microhybrid and a modern nanohybrid composite is twofold: Microhybrid composites serve as a well-established benchmark in restorative dentistry due to their proven mechanical properties and clinical history. They also represent the forefront of material technology, offering enhanced aesthetics and potential improvements in wear resistance [1,2,3].

## 2. Materials and Methods

**Figure 1:**
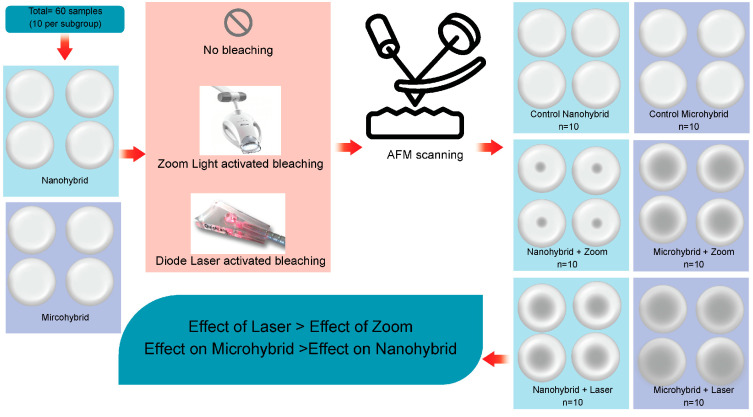
Schematic illustration of the study design (Arrows indicate the increase in surface roughness—larger arrows correspond to higher roughness values).

Two restorative materials with the same A2 shade were utilized in this investigation (Table 1): a nanohybrid composite resin material (Herculite XRV Kerr Italia S.r.l., Salerno, Italy) and a microhybrid composite resin material (HerculiteUltraKerr Italia S.r.l., Salerno, Italy). Each material was further allocated into three subgroups (control, Zoom light-activated bleaching, and diode laser bleaching). A schematic illustration summarizes the study design, showing the sample grouping, bleaching procedures, and subsequent surface roughness evaluation by AFM (Figure 1).

The bleaching agents used were as follows:

- Zoom Whitening + Zoom Gel (Philips Zoom, WA, USA): a light-activated bleaching system using 40% hydrogen peroxide gel.
- QuickLase Diode Laser + QuickLase Gel (QuickLase, Kent, UK): a diode laser-activated bleaching system (940 nm) with 35% hydrogen peroxide gel. For this study, the following materials, as presented in Table 1, were selected.

**Figure 2:**
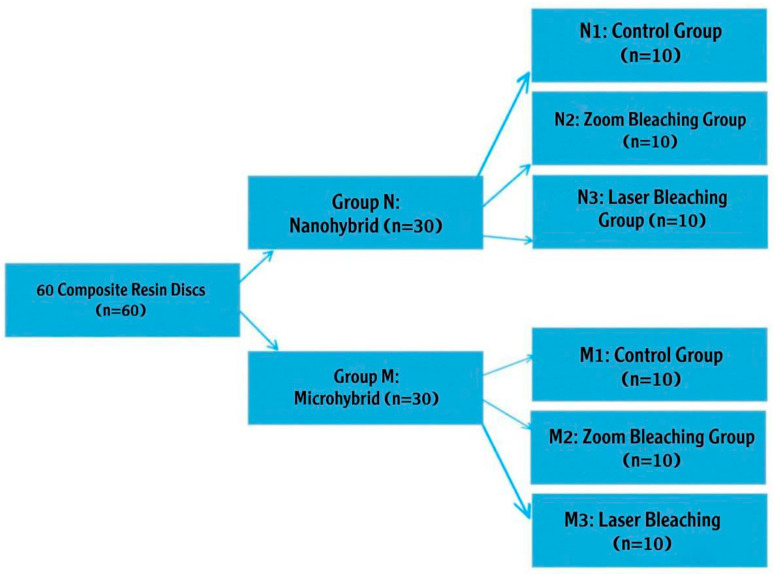
Distribution of composite resin disks among the experimental subgroups. Arrows indicate the grouping and sample allocation.

After completing the composite resin disk (CRD) preparation, the disks were categorized into two groups according to the type of resin composite restorations and bleaching protocol [Figure 2].

Group N: 30 CRDs for the nanohybrid composite material; group M: 30 CRDs for the microhybrid composite material. Each group was then subdivided according to the bleaching protocol into three subgroups (10 CRDs each):

- N1 and M1 (control subgroups): no bleaching was applied.
- N2 and M2 (zoom subgroups): bleached using Philips Zoom light with zoom bleaching material (light activation technique).
- N3 and M3 (laser subgroups): bleached using QuickLase diode laser (940 nm) with QuickLase bleaching material (laser activation technique).

### 2.1. Preparation of Samples

The required sample size was calculated using G*Power software version 3.1 (Heinrich Heine University, Düsseldorf, Germany). The effect size (Cohen’s f) was estimated based on the results of a previous study [15] as f = 0.8, with an alpha (α) level of 0.05 and a desired power (1 − β) of 0.80. The analysis indicated that a total sample size of 60 was required. This was allocated as 10 specimens per group to ensure adequate power to detect statistically significant differences.

A total of 60 composite resin disks (CRDs) were constructed from a Teflon mold and divided into two main groups (*n* = 30/group) based on the types of composite resin restorations. The dimension of the CRDs was 10 mm × 4 mm. A glass slab was overlaid with a microscopic glass slide. These dimensions ensured sufficient surface area for AFM scanning and adequate curing depth [16].

**Figure 3:**
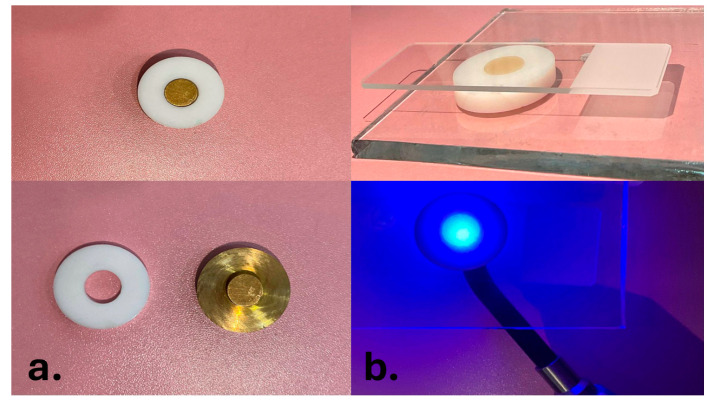
(**a**) Custom-made Teflon mold with metallic plunger. (**b**) Teflon mold positioned between a glass slab and microscope glass during light-curing.

**Figure 4:**
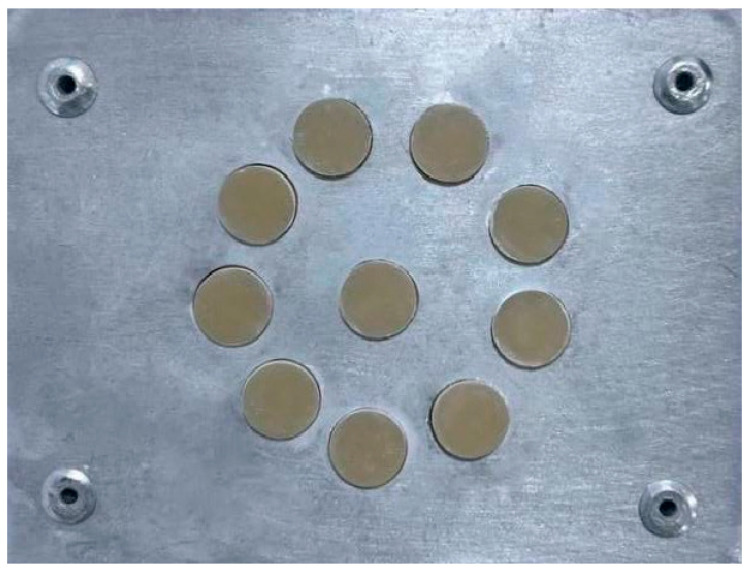
Standardized arrangement of composite resin samples within a metallic mold after preparation and before surface polishing.

Following placement of the resin composite material in the Teflon mold, the CRDs were then light-cured from the top using LED (Elipar S10, 3M ESPE, Düsseldorf, Germany) for 40 s (20 s from above and another from below the specimen) with intimate contact with the glass slab to minimize the curing distance, as shown in Figure 3. The intensity of the LED curing unit was verified with a radiometer and measured at 2000 mW/cm^2^. Digital calipers were used to measure the thickness of each sample. After the composite resin disks were prepared, specimens were placed in a custom-made metallic disk to ensure stable positioning during polishing. The metallic support provided a rigid and uniform backing, which minimized the vibration and movement of the specimens, thereby preventing surface irregularities and ensuring reproducibility of the polishing, as shown in Figure 4. Polishing of all specimens was standardized by using a single operator throughout the process. A contra-angle handpiece (Sof-Lex™ Contouring and Polishing Disks Kit, 3M ESPE, St. Paul, MN, USA) was employed at a constant low rotational speed recommended by the manufacturer. Each specimen was polished for 10 s using fine and superfine disks under uniform, mild pressure. Mild pressure was defined as light hand pressure just sufficient to maintain contact between the polishing disk and the specimen surface without causing deformation or excessive removal of material. To control and maintain consistency, the operator was calibrated in a pilot trial prior to the study. After polishing, all specimens were carefully inspected using a dental loupe (×2.5 magnification) to check for any surface defects such as cracks, voids, or irregularities. Specimens presenting visible defects were excluded and replaced with newly fabricated ones to ensure uniformity and reliability of the tested samples. All specimens were subjected to 10,000 thermocycles between 5 °C and 55 °C using a thermocycling machine equipped with an STC-1000 digital temperature controller and cycle (custom-made device, Mosul, Iraq) [17]. After fabrication, all disks were coded and then randomly assigned to the control, zoom bleaching, or laser bleaching subgroups (n = 10 per subgroup). This procedure ensured that each specimen had an equal chance of being allocated to any subgroup, thereby minimizing selection bias.

### 2.2. Bleaching Technique

Zoom bleaching was performed using Philips Zoom Whitespeed 40% hydrogen peroxide gel. Approximately 0.5 mL of gel was applied in a 2–3 mm uniform layer on each specimen surface using a graduated probe to ensure uniformity across all specimens, activated by the Zoom LED light for 15 min, and then removed and replaced with fresh gel for three consecutive cycles, with 1-min intervals between cycles, according to the manufacturer’s instructions.

Laser bleaching was carried out with QuickLase diode laser (940 nm) using QuickLase bleaching gel containing 35% hydrogen peroxide. Approximately 0.5 mL of gel was applied in a 2–3 mm layer, activated by the laser in two cycles of 30 s each, and left on the surface for 8 min after the second activation before being suctioned, rinsed, and dried. After bleaching, all composite resin disks (CRDs) were rinsed thoroughly with distilled water to remove residual bleaching agents and then dried using oil-free air.

The surface quality of a sample is quantified by measuring roughness, which shows the fissures, streaks, or traces resulting from a particular process of working or finishing/polishing. The profile of these traces was described using the following parameters: Sa (arithmetic mean height − average surface roughness (nm)), Sq (root mean square height − more sensitive to extreme values (nm)), and Sz (maximum height − total range (peak + valley) (nm)).

### 2.3. Measuring the Surface Roughness

The surface roughness of the composite resin disks was evaluated using Atomic Force Microscopy (CoreAFM, Nanosurf AG, Liestal, Switzerland). Each specimen was fixed on the sample holder and scanned in tapping mode under ambient laboratory conditions. A scanning area of 20 × 20 μm^2^ was selected for each sample at the center of the surface. For each specimen, three scans were obtained at different surface regions, and the mean values were calculated. The surface roughness parameters assessed were the arithmetic mean height (Sa), root mean square height (Sq), and maximum height (Sz), expressed in nanometers (nm). Data were recorded and exported using the instrument’s analysis software, The surface analysis was performed using Nanosurf® Easyscan software, version 2.1 (Nanosurf AG, Liestal, Switzerland).

### 2.4. Statistical Analysis

Statistical analysis was performed using SPSS^®^ version 25. The normality of distributions was assessed using the Shapiro–Wilk test. Differences between two independent variables were assessed using the Student’s *T*-test for independent samples. A *p*-value < 0.05 was considered statistically significant.

Based on the aim of the study, the following null hypotheses were tested:

**H1:**  *Neither bleaching protocol (zoom or laser) will significantly alter the surface roughness parameters (Sa, Sq, Sz) compared to an unbleached control within each composite type.*

**H2:**  *There will be no significant difference in post-bleaching surface roughness between nanohybrid and microhybrid composites for any given bleaching protocol.*

**H3:**  *There will be no significant difference in surface roughness between zoom-activated and laser-assisted bleaching protocols within the same composite type.*

It is anticipated that the findings of this study will provide valuable insights into the interactions between contemporary bleaching techniques and composite resin surfaces. This investigation may contribute to the broader knowledge in dental material science by offering evidence-based data that could aid clinicians in selecting restorative materials and bleaching protocols that are most compatible, thereby potentially enhancing the aesthetic outcomes and longevity of dental restorations.

## 3. Results

This study evaluated the surface roughness parameters (Sa) of microhybrid and nanohybrid composites subjected to different bleaching protocols. The values were recorded and statistically analyzed. The results are summarized in Table 2.

**Figure 5:**
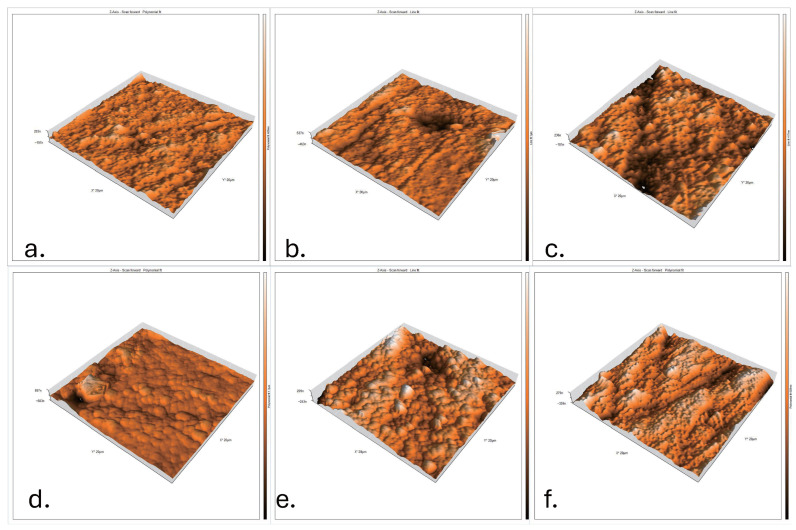
Atomic Force Microscopy (AFM) pseudo-color and 3D surface topography images (scan area: 20 × 20 μm^2^).

All AFM images presented in Figure 5 were acquired under standardized conditions, using an identical scan size of 10 μm × 10 μm and fixed magnification parameters. This uniformity ensured accurate and reliable comparison of surface topography among the different experimental groups.

(a) N1: Untreated nanohybrid composite surface showing a smooth and compact topography with minimal irregularities.
(b) N2: Nanohybrid composite after zoom bleaching, with moderate roughness and irregular elevations/depressions indicating resin matrix alteration.
(c) N3: Nanohybrid composite after laser bleaching, exhibiting pronounced peaks and valleys with extensive surface degradation.
(d) M1: Control microhybrid composite surface, relatively smooth but with more visible micro-filler particles compared to Nano Control.
(e) M2: Microhybrid composite after zoom bleaching, showing higher roughness with exposed filler–matrix boundaries due to oxidative stress.
(f) M3: Microhybrid composite after laser bleaching, presenting the most disrupted surface, severe heterogeneity, and significant elevation differences.

These images confirm that laser bleaching induces the most aggressive surface damage, especially in microhybrid composites, followed by zoom bleaching, while control samples preserve smoother topography. This qualitative AFM analysis supports the quantitative roughness data (Sa) obtained in this study.

### 3.1. Micro- vs. Nano-Surface Roughness After Bleaching

The statistical comparison between nanohybrid and microhybrid composites is summarized in Table 3.

**Figure 6:**
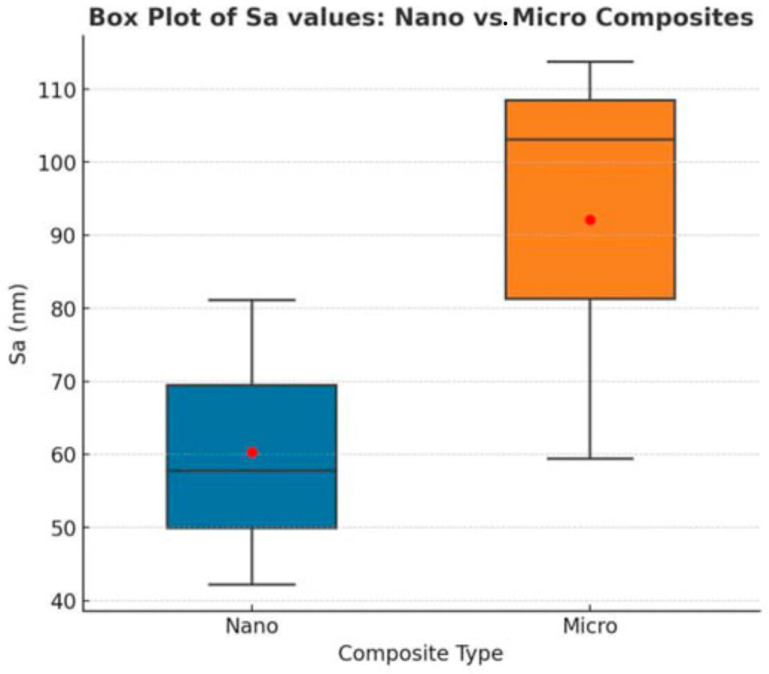
Box plot illustrating the arithmetic mean height (Sa, nm) of nanohybrid and microhybrid composite resins under different conditions (control, zoom bleaching, laser bleaching). The central line inside each box represents the median value, while the red dot indicates the mean. The box boundaries represent the interquartile range (IQR), and the whiskers extend to the minimum and maximum values. Individual data points (red dots) correspond to the mean Sa values of each subgroup. Nanohybrid composites (Nano Control, Nano Zoom, Nano Laser) exhibited consistently lower roughness compared to microhybrid composites (Micro Control, Micro Zoom, Micro Laser).

Figure 6 illustrates the comparison between nanohybrid and microhybrid composites in terms of surface roughness values (Sa) under various bleaching conditions.

### 3.2. Zoom vs. Laser: Within-Group Roughness Comparison

The statistical differences in surface roughness between the two bleaching methods (Zoom and Laser) for both nanohybrid and microhybrid composites are summarized in Table 4.

**Figure 7:**
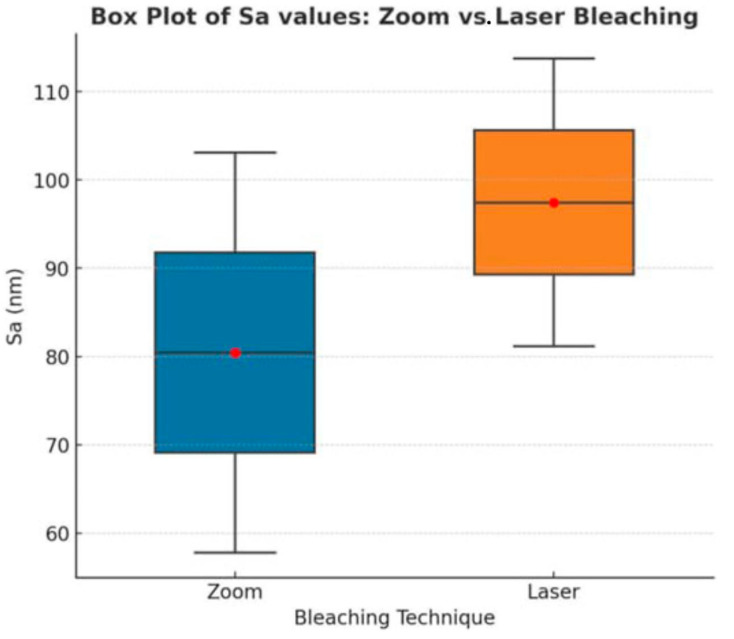
Box plot comparing the arithmetic mean height (Sa, nm) between zoom (light-activated) and laser (diode-activated) bleaching techniques. The median is represented by the central line in each box, and the red dot indicates the mean value. The boxes display the interquartile range (IQR), with whiskers extending to the minimum and maximum. red dots represent the mean Sa values of each subgroup (Nano Zoom, Micro Zoom, Nano Laser, Micro Laser). Both techniques increased surface roughness compared to controls, with laser bleaching showing a more pronounced effect overall.

Figure 7 shows a box plot comparing the surface roughness (Sa) values between Zoom and Laser bleaching techniques for both composite types.

## 4. Discussion

The findings led to the rejection of H1 and H2 in both composite types. However, H3 was rejected only in the nanohybrid group; in the micro-hybrid group, the difference between bleaching protocols was not statistically significant (*p* = 0.765), demonstrating that both bleaching protocols significantly increased surface roughness compared to unbleached controls, that a significant difference in post-bleaching roughness existed between composite types, and that a significant difference was found between bleaching methods within the nanohybrid group.

The most salient finding is the superior resistance of nanohybrid composites to bleaching-induced surface degradation compared to microhybrid composites, a result consistent with previous research indicating better surface integrity in nanofilled materials after chemical challenging. This disparity can be attributed to fundamental differences in their microstructure. Nanohybrid composites incorporate a combination of prepolymerized fillers, nano-sized silica particles, and submicron barium glass fillers. This sophisticated filler system creates a more homogeneous and densely packed matrix with a higher filler load [18]. The nanofillers effectively occupy the interstices between larger particles, resulting in a smoother initial surface and a more stable, less permeable filler–resin interface that is more resistant to chemical erosion by hydrogen peroxide [19]. Conversely, the larger filler particles and potentially less homogeneous matrix of the microhybrid composite provide a more pronounced pathway for the oxidizing agent to infiltrate and degrade the organic resin matrix (Bis-GMA, TEGDMA), weakening the encapsulation of filler particles and making them more susceptible to being plucked out [20], thus creating a rougher surface topography.

The comprehensive AFM analysis, which evaluated the parameters Sa, Sq, and Sz, provides a multi-faceted and clinically relevant understanding of the surface alterations. The arithmetic mean height (Sa) offers a general overview of average roughness, and its increase confirmed that both bleaching treatments roughened the surfaces, supporting previous findings that bleaching agents alter surface topography, similar to surface alterations described in other studies evaluating bleaching effects [21,22].

The trends of parameters—Sa—were consistent: control < zoom-bleached < laser-bleached, and nanohybrid < microhybrid within each treatment condition. This consistent hierarchy validates the findings and provides a complete topographic profile of the damage.

The difference between the two bleaching techniques is fundamentally rooted in their mechanism of action. The Zoom LED system primarily provides photochemical activation, accelerating the breakdown of the hydrogen peroxide gel into free radicals that oxidize and degrade the organic structure [23]. The photothermal contribution of the diode laser is a hypothetical yet plausible explanation for the higher roughness observed; however, this study did not include temperature measurements or independent controls to confirm this causal mechanism. We speculate that the heat may cause transient softening of the resin matrix [24].

Furthermore, the heat may cause transient softening of the resin matrix, making it more vulnerable to chemical degradation and mechanical alteration, a phenomenon observed in studies of laser effects on dental materials [24,25]. This synergistic chemo-thermal effect explains why the laser protocol consistently resulted in higher values of AFM parameters (Sa) indicating a more severe and irregular surface degradation compared to the purely photochemical zoom method.

The potential clinical implications of these findings are substantial. Based on the established correlation between surface roughness and clinical behavior, surfaces with roughness values (Sa) exceeding a threshold of approximately 0.2 µm are theoretically known to promote plaque adhesion. The values measured in this study far exceed this threshold, suggesting that polishing composite restorations after any in-office bleaching procedure may be essential [26]. The values measured in this study, particularly for the bleached microhybrid groups, far exceed this threshold. Therefore, polishing composite restorations after any in-office bleaching procedure is not merely advisable but essential to restoring a plaque-resistant surface. From a material selection perspective, the use of nanohybrid composites in patients who may undergo future bleaching treatments is strongly recommended to mitigate these adverse effects.

Clinically, the observed increase in surface roughness after bleaching may predispose restorations to plaque accumulation, staining, and decreased longevity. Therefore, repolishing after bleaching and preferring nanohybrid composites for patients undergoing bleaching may enhance clinical outcomes.

These findings should be interpreted with caution, as they represent in vitro data. No in-mouth validation such as biofilm adhesion or staining evaluation was performed in this study. Therefore, the clinical recommendations suggested here are potential implications rather than definitive conclusions.

Future research should build upon these findings by correlating these topographical changes with functional clinical measures, such as quantitative biofilm adhesion studies and assessments of abrasion resistance. Furthermore, investigating the effect of different laser parameters—including wavelength, power, and exposure duration—on composite surface properties would help refine clinical protocols to minimize iatrogenic damage. Finally, long-term clinical trials are ultimately necessary to monitor the performance of different composite types following various bleaching protocols in the dynamic oral environment, thereby translating these robust in vitro results into evidence-based clinical practice. Such research directions would address the current gap between laboratory findings and clinical applications in aesthetic dentistry.

Limitations: This was an in vitro study without clinical validation (e.g., biofilm or staining tests). Future research should investigate long-term clinical outcomes, biofilm adhesion, and temperature effects during laser bleaching.

## 5. Conclusions

This study demonstrated that:

- Both Zoom and laser bleaching increased composite surface roughness.
- Laser bleaching produced more pronounced alterations overall.
- Nanohybrid composites showed better resistance compared to microhybrid composites.
- Clinical caution is advised: polishing after bleaching is recommended.
- Findings are in vitro and should be validated in clinical settings.

## Figures and Tables

**Table 1:** Materials used in this study.

Material Type	Name	Manufacturer	Composition/Specifications	Lot No.	Application Procedure
Composite Resin (Microhybrid)	HerculiteXRV (Microhybrid Composite)	Kerr Italia S.r.l., Salerno, Italy	Bis-GMA, TEGDMA, barium glass and silicon dioxide fillers, additives, stabilizers, catalysts	11,023,474	Fabricated into 10 × 4 mm disks; light-cured 20 s per side with LED unit; finished/polished before testing
Composite Resin (Nanohybrid)	HerculiteUltra (Nanohybrid Composite)	Kerr Corporation, Orange, CA, USA (or Kerr Italia S.r.l., Salerno, Italy)	Bis-GMA, TEGDMA, prepolymerized filler, silica nanofiller (20–50 nm), barium submicron fillers (0.6 µm), TiO_2_, pigments	11,023,471	Same as above
Bleaching Agent (Light-activated)	Zoom Gel 40% HP	Philips Oral Healthcare, Bothell, WA, USA	Hydrogen peroxide 40% concentration	3,402,981	Apply ~2–3 mm layer; activate with Zoom LED for 15 min; remove gel; repeat ×3 with 1 min intervals; rinse and dry (per IFU)
Bleaching Agent (Laser-activated)	QuickLase Gel 35% HP	QuickLase Ltd., Canterbury, Kent, UK	Hydrogen peroxide 35% concentration; vehicle (water/glycerin), thickener, stabilizers/buffer	101,101	Apply ~2–3 mm layer; activate with 940 nm diode laser for two cycles × 30 s; leave gel ~8 min after second cycle; suction, rinse and dry (per IFU)

Abbreviations: Bis-GMA, bisphenol A-glycidyl methacrylate; TEGDMA, triethylene glycol dimethacrylate; TiO_2_, titanium dioxide; HP, hydrogen peroxide; LED, light-emitting diode.

**Table 2:** Summary of mean surface roughness (Sa, nm) values ± standard deviation (SD) and standard error (SE) for nanohybrid (N) and microhybrid (M) composite groups after different bleaching protocols (control, Zoom, and laser).

Group	Sa Mean	Sa SD	Sa SE
N1	42.18	6.62	2.09
N2	57.77	13.88	4.39
N3	81.15	22.06	6.98
M1	59.43	9.33	2.95
M2	103.12	19.25	6.09
M3	113.74	36.16	11.43

**Table 3:** Statistical comparison of surface roughness (Sa) values between nanohybrid and microhybrid composites subjected to zoom and laser bleaching.

Groups		N	Mean	Std. Deviation	Test Value	*p*-Value
Zoom	Nano	10	57.7720	13.87759	6.042	0.0001 **
	Micro	10	103.1150	19.25045		
Laser	Nano	10	78.1270	23.29051	2.583	0.019 *
	Micro	10	106.1600	25.21317		

* Significant difference at *p* < 0.05. ** Highly significant difference at *p* < 0.001.

**Table 4:** Statistical comparison of surface roughness (Sa) values between zoom and laser bleaching techniques within nanohybrid and microhybrid composite groups.

Groups		N	Mean	Std. Deviation	Test Value	*p*-Value
Nano	Zoom	10	57.7720	13.87759	2.374	0.032 *
	Laser	10	78.1270	23.29051		
Micro	Zoom	10	103.1150	19.25045	0.304	0.765
	Laser	10	106.1600	25.21317		

* Statistically significant at *p* < 0.05.

## Data Availability

The original contributions presented in this study are included in the article. Further inquiries can be directed to the corresponding author.

## Abbreviations

The following abbreviations are used in this manuscript:

AFM	Atomic Force Microscopy
CRD	Composite Resin Disk
Sa	Arithmetic Mean Height
Sq	Root Mean Square Height
Sz	Maximum Height
LED	Light-Emitting Diode
nm	Nanometer
µm	Micrometer
Bis-GMA	Bisphenol A-Glycidyl Methacrylate
TEGDMA	Triethylene Glycol Dimethacrylate
USA	United States of America
